# Risk Assessment and Prediction of Hepatocellular Carcinoma in Noncirrhotic Metabolic Dysfunction-Associated Steatotic Liver Disease

**DOI:** 10.3390/ijms27073241

**Published:** 2026-04-02

**Authors:** Emilie K. Mitten, Piero Portincasa, György Baffy

**Affiliations:** 1 Department of Medicine, Division of Gastroenterology, Massachusetts General Hospital, Harvard Medical School, Boston, MA 02115, USA; emitten@bwh.harvard.edu; 2 Division of Internal Medicine, Department of Precision and Regenerative Medicine, University ‘Aldo Moro’ Medical School, 70121 Bari, Italy; piero.portincasa@uniba.it; 3 Department of Medicine, Division of Gastroenterology, Hepatology and Endoscopy, Brigham and Women’s Hospital, Harvard Medical School, Boston, MA 02115, USA; 4 Department of Medicine, Section of Gastroenterology, VA Boston Healthcare System, 150 South Huntington Ave., Room 6A-46, Boston, MA 02130, USA

**Keywords:** steatotic liver disease, metabolic burden, machine learning, cluster analysis, noncirrhotic, hepatocarcinogenesis, surveillance

## Abstract

Metabolic dysfunction-associated steatotic liver disease (MASLD) has emerged as a leading driver of hepatocellular carcinoma (HCC) worldwide. A substantial proportion of MASLD-related HCC arises in the noncirrhotic liver, highlighting critical gaps in current surveillance strategies that rely primarily on fibrosis stage to define risk. Although the annual incidence of HCC in noncirrhotic MASLD is low and does not justify universal surveillance, the extraordinary global prevalence of MASLD translates into a considerable absolute burden of cancer. Accumulating evidence demonstrates that HCC risk in MASLD is modulated not only by histologic severity but also by metabolic comorbidities, particularly type 2 diabetes mellitus, which can significantly amplify cancer risk even in pre-cirrhotic stages. From both clinical and health economic perspectives, these observations underscore the need for more complex and targeted surveillance approaches. This review synthesizes current epidemiologic data, metabolic and histologic modifiers of HCC risk, emerging biomarkers, and predictive models in MASLD, with a focus on noncirrhotic disease. We discuss how integrated, precision-based risk assessment may identify high-risk MASLD subgroups and enable targeted, cost-effective surveillance strategies to mitigate the growing burden of MASLD-associated HCC.

## 1. Introduction

Metabolic dysfunction-associated steatotic liver disease (MASLD) is one of the most prevalent chronic liver diseases worldwide and a leading contributor to hepatocellular carcinoma (HCC) [1,2,3]. HCC remains the only major cancer for which mortality has not substantially improved over the past decade, in contrast to trends observed for most other malignancies [4]. Traditionally, cirrhosis has been considered the principal substrate for hepatocarcinogenesis, and indeed, approximately 80% of patients with HCC have underlying cirrhosis across etiologies [5,6]. However, MASLD challenges this paradigm and raises critical questions regarding early prediction and risk stratification [7].

An important conundrum in the natural history of MASLD is that HCC frequently develops in the noncirrhotic liver, accounting for up to 50% of all MASLD-related HCC cases in some series [8,9,10,11]. These observations suggest that MASLD may involve distinctly different mechanisms of oncogenesis compared with chronic liver diseases of other etiologies. Early pathogenic events, such as metabolic dysregulation, lipotoxicity, and low-grade inflammation, may directly promote liver tumor formation [12,13,14]. For example, in an Italian multicenter prospective observational study analyzing the features of MASLD- and HCV-associated HCC, only 54% of MASLD-HCC patients had established cirrhosis at the time of diagnosis with HCC compared to 97% of those with HCV-HCC [15], underscoring a major limitation of MASLD-related HCC surveillance strategies that rely predominantly on the presence of cirrhosis to define eligibility.

**Figure 1:**
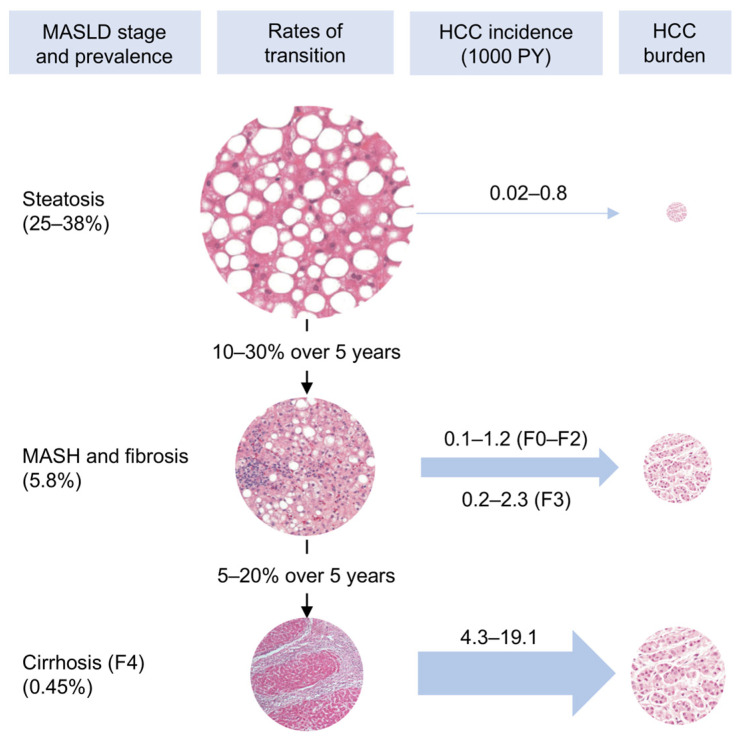
Population burden of MASLD-associated HCC across disease stages. Schematic representation of metabolic dysfunction-associated steatotic liver disease (MASLD) stages in the adult population, with corresponding hepatocellular carcinoma (HCC) incidences expressed per 1000 person-years (PY). Although HCC risk rises substantially with advancing fibrosis and cirrhosis, the high prevalence of noncirrhotic MASLD (including steatosis and steatohepatitis) results in a substantial cumulative contribution to the overall HCC burden, accounting for a sizeable proportion of cases. Estimates of disease prevalence, HCC incidence, and transition rates are derived from published reports [16,17,18,20,24,25,26,27,28,29,30,31,32,33,34]. Circle sizes are illustrative and not drawn to scale. F4, stage 4 fibrosis; HCC, hepatocellular carcinoma; MASH, metabolic dysfunction-associated steatohepatitis; MASLD, metabolic dysfunction-associated steatotic liver disease; PY, person-year.

The primary barrier to extending HCC surveillance in MASLD beyond patients with established cirrhosis is the very low annual incidence of HCC in those with “simple steatosis” (i.e., hepatic steatosis without necroinflammation or fibrosis, also termed MASL), estimated at 0.02% to 0.08% [16,17,18]. Even among patients with pre-cirrhotic metabolic dysfunction-associated steatohepatitis (MASH; F0–F3), annual HCC incidence ranges from 0.1% to 0.7% [17,18,19]. These rates fall below accepted cost-effectiveness thresholds for universal HCC surveillance, historically ~1.5% per year, although more recent analyses suggest that a lower threshold of approximately 0.4% per year may still be cost-effective when ultrasound with alpha fetoprotein (AFP) is used in patients with cirrhosis [20,21,22]. Consequently, universal surveillance in noncirrhotic MASLD is not economically justified. However, given the extraordinary global prevalence of MASLD, even low HCC incidences translate into a considerable absolute number of noncirrhotic HCC cases, representing a substantial and growing public health burden [23] (Figure 1).

**Figure 2:**
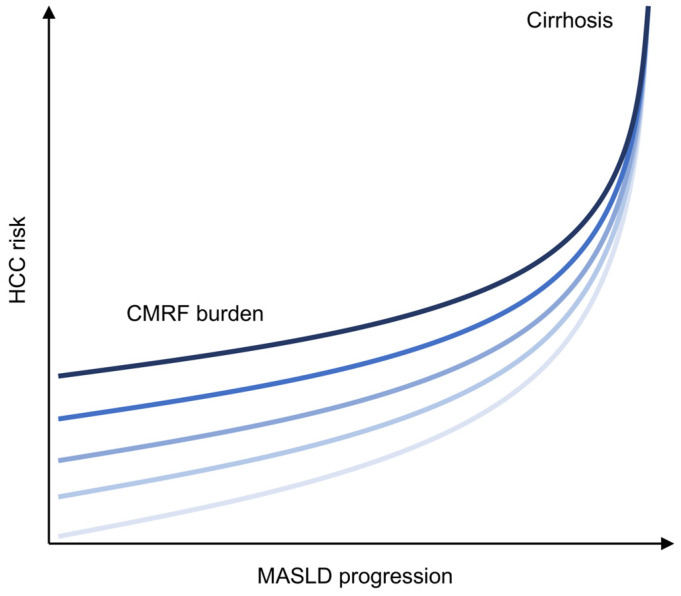
Transition from metabolic to fibrosis-driven HCC risk in MASLD. Schematic representation of HCC risk across MASLD progression. MASLD is defined by hepatic steatosis plus at least one of five cardiometabolic risk factors (CMRF; i.e., abdominal obesity, high blood pressure, high triglycerides (TGs), low high-density lipoprotein (HDL) cholesterol, and impaired glucose tolerance) [36]. In early disease stages, increasing metabolic burden (e.g., the increasing number of CMRFs from 1 to 5, illustrated here by progressively darker blue lines) is a primary determinant of HCC risk in noncirrhotic MASLD, resulting in distinct risk trajectories. With advancing fibrosis, the influence of metabolic burden diminishes, and cirrhosis emerges as the dominant driver of HCC risk, leading to convergence toward a uniformly high-risk state.

HCC risk in MASLD increases progressively with histologic severity. In a large population-based Swedish cohort of 8892 adults with histologically defined MASLD followed for a median of 13.8 years, HCC incidence increased monotonically across simple steatosis, nonfibrotic MASH, noncirrhotic fibrosis, and cirrhosis (0.8, 1.2, 2.3, and 6.2 per 1000 person-years (PY), respectively; *p* for trend <0.01) [18]. Importantly, type 2 diabetes mellitus (T2D) substantially amplified HCC risk at every stage (1.2, 2.9, 7.2, and 15.7 per 1000 PY, respectively) [18]. Individuals with MASLD and T2D who have additional traits associated with metabolic syndrome (hypertension, dyslipidemia, and obesity) may be at an even greater risk for HCC. A multicenter study examined over 270,000 patients with MASLD at Veterans Healthcare Administration (VHA) facilities and found that T2D was independently associated with HCC development (approximately 2.8-fold higher risk) after adjustment for demographic and metabolic factors [35]. Moreover, individuals with T2D plus two or three other metabolic traits exhibited a 5.55- to 8.63-fold greater risk for developing HCC [35]. These data indicate that fibrosis stage alone is insufficient to define HCC risk and that metabolic comorbidities can meaningfully modify the risk of hepatocarcinogenesis (Figure 2).

From a health economic perspective, a risk-stratified approach to HCC surveillance is therefore essential. Markov model-based simulations suggest that surveillance strategies become cost-effective when there is at least a 2-fold difference in HCC incidence between high- and low-risk groups [37]. This reinforces the need for robust biomarkers and predictive models that are able to identify subgroups of patients with noncirrhotic MASLD whose annual HCC risk approaches or exceeds accepted cost-effectiveness thresholds. Such precision risk stratification could justify medically indicated and economically sustainable surveillance, while avoiding unnecessary screening in the vast majority of patients at low absolute risk.

Importantly, even among patients with MASLD-related cirrhosis (where HCC surveillance is clearly recommended), screening uptake remains suboptimal and currently available modalities suffer from major limitations [38,39]. Ultrasound combined with AFP detects early-stage HCC with a sensitivity of only 63% [40,41]. Thus, improving early prediction and refining risk assessment is critical not only to determining who should be screened, but also to optimizing how surveillance is implemented.

In this review, we examine current evidence regarding early prediction and risk assessment for HCC in MASLD, with particular emphasis on the development of HCC in noncirrhotic disease. We discuss epidemiologic data, metabolic and histologic modifiers of risk, emerging biomarkers, and predictive models, and we explore how these tools may inform a targeted, cost-efficient surveillance strategy aimed at mitigating the growing burden of MASLD-related HCC.

## 2. Clinical Features for Risk Stratification of HCC in Noncirrhotic MASLD

Given that the overall annual incidence of HCC in noncirrhotic MASLD remains below established cost-effectiveness thresholds for universal surveillance, substantial efforts have focused on identifying combinations of clinical features that define higher-risk subgroups [21,42,43,44,45]. Recent large-scale cohort studies provide important insights into how demographic and metabolic factors refine risk of developing HCC in MASLD beyond fibrosis stage alone. Table 1 summarizes findings from English-language retrospective cohort studies of adults (*n* > 10,000) published within the past 10 years that report incidence of HCC in noncirrhotic MASLD.

### 2.1. Large Administrative Cohort Data

A recent retrospective cohort study utilized a large US administrative claims database (2007–2021) to better understand clinical risk factors, individually and in combination, for incident HCC [33]. Using ICD codes, the investigators identified 741,816 patients with steatotic liver disease (SLD) without significant alcohol use, viral hepatitis, other liver diseases, or prevalent HCC. Of these, 108,862 (14.7%) had cirrhosis and 632,954 (85.3%) did not. HCC incidence rates were estimated overall and stratified by risk factors, including sex, age, cirrhosis, T2D, chronic kidney disease (CKD), cardiovascular disease (CVD), non-liver cancers, smoking, and obesity [33]. Over 2,410,166 PY of follow-up, 1740 patients developed HCC, corresponding to an overall HCC incidence of 0.72 per 1000 PY. As expected, HCC incidence was markedly higher in the presence versus absence of cirrhosis (4.29 vs. 0.14 per 1000 PY). However, several non-fibrotic clinical features were independently associated with higher HCC incidence. Rates were higher among individuals aged ≥50 vs. <50 years (1.20 vs. 0.16 per 1000 PY), in males vs. females (0.95 vs. 0.52 per 1000 PY), and in those with T2D vs. those without (1.19 vs. 0.41 per 1000 PY). CKD, CVD, and non-liver cancers were also associated with substantially higher incidence rates (2.20, 1.89, and 3.90 per 1000 PY, respectively). Smoking conferred only a modest increase (0.80 vs. 0.70 per 1000 PY), while obesity was paradoxically associated with lower observed HCC incidence (0.58 vs. 0.81 per 1000 PY), a finding that contrasts with prior literature.

Further stratification by cirrhosis, sex, age, and T2D identified the highest-risk group as cirrhotic males >70 years of age with T2D (HCC incidence of 19.06 per 1000 PY) and the lowest-risk group as noncirrhotic females <40 years of age without T2D (0.04 per 1000 PY). Among individuals without cirrhosis, males >70 years of age with T2D had an HCC incidence of 1.36 per 1000 PY, which was significantly higher than the overall population incidence but still much lower compared to those with cirrhosis. Based on data from nearly three-quarters of a million patients with SLD and over 2.4 million PY of follow-up, this study provides precise and detailed estimates of HCC incidence rates for subgroups stratified not only on key factors such as age, sex, cirrhosis, and T2D, but also important comorbidities such as CVD, CKD, obesity, and smoking. Moreover, the findings highlight that clinical practice and public health guidelines should not rely solely on overall HCC incidence estimates, which may obscure clinically meaningful heterogeneity among subgroups of MASLD with different combinations of risk factors [33].

### 2.2. Familial and Shared Environmental Risk

Beyond individual clinical features, familial clustering studies suggest that shared genetic and environmental factors contribute to HCC risk in MASLD. In a nationwide Swedish multigenerational cohort including family members (relatives and spouses) of adults with biopsy-proven MASLD, first-degree relatives (FDRs) of individuals with MASLD had a higher HCC incidence than matched population comparators (13 vs. 8 per 100,000 PY; adjusted hazard ratio [aHR] 1.80, 95% CI 1.36–2.37) [46]. The risk of HCC development was further increased among FDRs of individuals with fibrosis or cirrhosis compared with those whose relatives had simple steatosis or non-fibrotic MASH (aHR 2.14, 95% CI 1.07–4.27). Although absolute risks were low, these data demonstrate familial aggregation of HCC.

Interestingly, spousal T2D emerged as an independent predictor of HCC (aHR 10.5, 95% CI 2.83–39.12), highlighting the potential importance of shared metabolic and lifestyle factors [46]. Supporting these findings, another study of caregivers (70% of whom were spouses) found that 79% of the participants had MASLD and 28% had suspected advanced fibrosis on transient elastography [49]. Together, these findings further emphasize the contribution of shared environmental exposures and metabolic traits in shaping clinical trajectories of MASLD including the development of HCC.

### 2.3. Steatosis Burden and HCC Risk in Noncirrhotic MASLD

While fibrosis severity remains a central determinant of HCC risk, emerging evidence indicates that hepatic steatosis, particularly when associated with substantial metabolic dysfunction, may independently contribute to hepatocarcinogenesis in noncirrhotic MASLD [50,51,52]. In this context, quantification of early steatosis, eventually with the help of artificial intelligence (AI)-supported ultrasonography [53], may improve patient risk stratification. Quantitative indices of steatosis have recently gained attention as potential tools for refining risk assessment beyond fibrosis alone. The use of controlled attenuation parameter (CAP) to estimate steatosis severity, either alone or in combination with the liver stiffness-based vibration-controlled transient elastography (VCTE), has shown promising but controversial findings in the prediction of HCC [54]. The use of the Fatty Liver Index (FLI), a validated surrogate marker for steatosis derived from waist circumference, body mass index (BMI), triglycerides, and gamma-glutamyl transferase (GGT) [55], also appears to hold promise in the prediction of HCC.

The utility of the FLI for HCC prediction was demonstrated in a large population-based study of 92,761 individuals with T2D, aged 40–79 years, who underwent two standardized health screenings between 2012 and 2015, with hepatic steatosis estimated using the FLI [48]. Participants were stratified by baseline FLI (<30, 30–59.9, ≥60) and by longitudinal changes between screenings. Incident HCC was ascertained using ICD codes and reimbursement data during follow-up from 2016 to 2020. Compared with individuals with FLI < 30, those with FLI 30–59.9 and FLI ≥ 60 had a 1.90-fold and 2.94-fold higher risk of HCC, respectively (both *p* < 0.01) [48]. Importantly, dynamic changes in steatosis burden were also associated with altered risk of HCC: an increase in FLI from <30 to ≥30 conferred a 2.10-fold higher risk (*p* < 0.01), whereas a reduction in FLI from ≥30 to <30 was associated with a 36% relative risk reduction (hazard ratio 0.64; *p* = 0.03). Notably, the protective association of FLI reduction became apparent after approximately three years, suggesting that sustained metabolic improvement may translate into meaningful oncologic benefit over time.

Although limited to a Korean T2D population and thus not universally generalizable, this study supports the clinical relevance of both the degree and trajectory of steatosis for HCC risk stratification in noncirrhotic MASLD [48]. While reduction in hepatic steatosis is biologically plausible as a strategy to lower HCC risk, direct evidence that steatosis improvement per se reduces incident HCC remains lacking. Observational data from bariatric surgery cohorts similarly suggest reduced HCC risk [56], but concurrent improvements in metabolic and fibrotic pathways preclude isolation of the specific contribution of steatosis reduction.

### 2.4. Clinical Risk Prediction Models: The HCC-RIFLE Score

Efforts to develop HCC risk prediction tools for noncirrhotic MASLD have led to models based on readily available clinical variables. The HCC-RIFLE (HCC RIsk in nonalcoholic Fatty Liver diseasE) model was derived from and internally validated in a nationwide Korean cohort and externally validated using a hospital-based cohort of patients with noncirrhotic MASLD patients [47]. The nationwide cohort included 409,088 patients ages 40–69 years with a diagnosis of MASLD (defined by a Hepatic Steatosis Index [HSI] of ≥36 and exclusion of other liver diseases) without cirrhosis or pre-existing HCC. Over a median follow-up of 10 years, 841 patients (0.21%) developed HCC, corresponding to an annual incidence of 0.21 per 1000 PY. In the external cohort of 8721 patients with MASLD diagnosed by ultrasound elastography and followed for a median of 9.2 years, 13 patients (0.15%) developed HCC, with a similar annual incidence of 0.19 per 1000 PY [47].

Using Cox proportional hazards modeling, six independent predictors were incorporated into the final model: age, sex, diabetes, BMI ≥ 25 kg/m^2^, alanine aminotransferase (ALT) elevation (>30 U/L in women, >34 U/L in men), and GGT > 41 U/L [47]. Risk scores ranged from 0 to 11 points, corresponding to annual HCC incidences of <0.1% (low-risk, 0–6 points), 0.1–0.2% (moderate-risk, 7–8 points), and >0.2% (high-risk, 9–11 points) [47]. Model discrimination was moderate, with AUROCs of 0.75 at 10 years in the derivation cohort and 0.84 in the validation cohort, and a c-index of 0.82 [47].

Notably, BMI ≥ 25 kg/m^2^ was associated with increased risk of HCC in this study [47], in contrast to the inverse association reported in a US administrative cohort [33]. These seemingly conflicting findings align with prior observations that patients with lean SLD may experience worse outcomes than those with overweight or obesity [57]. Although these discrepancies warrant further investigation, the overall positive association between obesity, steatosis, and HCC risk is supported by broader epidemiologic evidence linking obesity to hepatocarcinogenesis [6]. While the HCC-RIFLE model may improve risk stratification in noncirrhotic MASLD, its generalizability remains uncertain and requires further validation across diverse populations and healthcare settings.

## 3. Use of Noninvasive Fibrosis Tests to Define HCC Risk in Noncirrhotic MASLD

Non-invasive tests (NITs), including serum-based tests, such as the Fibrosis-4 Index (FIB-4) and NAFLD fibrosis score (NFS), as well as liver stiffness-based methods such as VCTE, are well validated for fibrosis staging in MASLD and are widely utilized in routine clinical practice [58,59,60]. These tests have also demonstrated predictive value for HCC, particularly in cirrhotic MASLD [6] and potentially in noncirrhotic disease [61]. Similar to their use in fibrosis assessment, NITs are well suited for serial application, allowing for dynamic reassessment of HCC risk over time.

### 3.1. Serum-Based NITs

Elevated FIB-4 scores, both at baseline and longitudinally, have been linked to increased HCC risk compared with persistently low scores [62]. Notably, dynamic changes in FIB-4 appear more reliable than single time point measurements for predicting severe liver outcomes in MASLD [62,63,64,65]. In noncirrhotic MASLD, a FIB-4 ≥ 1.45 and NFS ≥ −1.455 were associated with 14-fold and 5.6-fold higher HCC risk, respectively, over 7.5 years of follow up [66]. In a large cohort of 202,319 US veterans with MASLD, longitudinal changes in FIB-4 were independently associated with incident cirrhosis and HCC. Among 473 patients who developed HCC, the incidence was 0.28 per 1000 PY (95% CI 0.26–0.30). A persistently high FIB-4 (>2.67 at baseline and at 3 years) conferred more than a 50-fold higher risk of developing HCC when compared to persistently low FIB-4 values (<1.45) [62]. These findings support the integration of simple, serial NITs such as FIB-4 into clinical decision-making strategies to identify subgroups in the MASLD population that could benefit from HCC surveillance.

In a multinational cohort of 613 adults with MASLD-related HCC (33% female) diagnosed between 2008 and 2023 with a mean follow-up duration of 2.65 years, 38% had no known cirrhosis at diagnosis [67]. Among those with noncirrhotic HCC, more than one-quarter had low FIB-4 scores, suggesting that current FIB-4-based clinical pathways may miss a substantial subset of at-risk individuals requiring further evaluation [67]. Furthermore, FIB-4 may be less reliable in men and in individuals with obesity and T2D [68,69], raising concerns about its sensitivity in metabolically high-risk populations. While low FIB-4 scores are generally associated with a very low risk of HCC, data on their distribution across fibrosis stages (F0/F1 vs. ≥F2) among patients who develop HCC remain limited [17,20].

A recent systematic review of 20 cohort studies (11 included in the meta-analysis) evaluated serum-based NITs for HCC risk stratification in noncirrhotic MASLD [61]. Diagnosis of MASLD was based on liver biopsy (8 studies), ultrasonography (5 studies), liver biopsy or radiographic imaging (4 studies), and ICD codes or laboratory data (3 studies), such as elevated ALT with negative viral hepatitis serologies. The NITs in these studies included FIB-4 (10 studies), aspartate aminotransferase (AST) to platelet ratio index (APRI) (7 studies), NFS (7 studies), and the BMI, AST/ALT Ratio, and Diabetes (BARD) score (7 studies).

Pooled adjusted risk ratios for incident HCC were 15.81 (95% CI 6.42–38.96), 4.08 (95% CI 1.25–13.30), and 4.13 (95% CI 1.61–10.61) for FIB-4, APRI, and NFS, respectively. The BARD score was not significantly associated with HCC (RR 1.46; 95% CI 0.73–2.92). Optimal cutoffs (AUROCs > 0.80) were >2.06, >0.65, and >0.51 for FIB-4, APRI, and NFSs, respectively, corresponding to HCC incidences of 3.38, 3.24, and 9.44 per 1000 PY. Cost-effectiveness thresholds (15 per 1000 PY) were reached at FIB-4 ≥ 5.91 and NSF ≥ 2.85 [61], values that likely reflect advanced fibrosis. In a subgroup of biopsy-confirmed noncirrhotic MASLD from 6 studies, pooled unadjusted and adjusted risk ratios for incident HCC with FIB-4 were 6.45 (95% CI 3.30–12.61; *n* = 1866, 2.7% HCC) and 3.16 (95% CI 0.43–23.04; *n* = 417, 2.4% HCC), respectively, with no significant differences based on MASLD diagnostic modality [61]. Collectively, these findings reinforce the utility of serum-based NITs as scalable tools for HCC risk assessment in noncirrhotic MASLD.

### 3.2. Liver Stiffness Measurement

Elevated baseline liver stiffness measurements (LSM) by VCTE have been associated with an increased risk of HCC [70,71,72]. Similarly, longitudinal increases in LSM have been linked to higher HCC risk, primarily in patients with cirrhosis [73]. More recently, attention has shifted toward noncirrhotic MASLD.

In a retrospective analysis of the Veterans Analysis of Liver Disease (VALID) cohort, patients with MASLD underwent transient elastography between 2014 and 2023 [74]. Among 30,414 participants (69,974 PY), each 5 kPa increase in baseline LSM was associated with an 18% higher risk of HCC. In a subgroup without cirrhosis or clinically significant portal hypertension (CSPH), men with T2D and LSM ≥ 10 kPa had an HCC incidence of 0.46 per 100 PY, approaching cost-effectiveness thresholds for HCC surveillance in this population [74]. These findings suggest that patients with LSM ≥ 10 kPa and T2D may represent a high-risk subgroup of noncirrhotic MASLD who could benefit from HCC surveillance. However, it remains uncertain whether this subgroup indeed represents noncirrhotic disease. The use of ICD codes and VCTE thresholds to exclude cirrhosis may have resulted in misclassification. Moreover, progression of fibrosis to cirrhosis over the follow-up period (up to 9 years) may have further influenced these findings [75].

Overall, while VCTE-derived LSM provides a useful surrogate of fibrosis and correlates with HCC risk, its direct applicability to noncirrhotic MASLD remains uncertain, as potential misclassification of advanced disease as well as distinct oncogenic pathways may limit its predictive specificity in this setting.

## 4. Studies on Genetic Predisposition to HCC in MASLD

Genetic factors play a significant role in adverse liver outcomes in MASLD [76]. Among these, *PNPLA3* variants are the most extensively studied and are strongly associated with steatosis, fibrosis, and HCC, including in noncirrhotic disease, supporting their relevance for early risk stratification [77,78]. Early genome-wide association studies (GWAS) identified the rs738409 (I148M) variant as a key determinant of hepatic fat accumulation and inflammation [79], with subsequent studies linking increasing liver fat burden to higher risks of cirrhosis and HCC [50,51,52]. These findings suggest that carriers of *PNPLA3* risk variants may benefit from early identification and targeted metabolic interventions.

Additional variants in *FTO*, *GCKR*, *MBOAT7*, *MTTP*, *PPARG*, *SERPINA1*, and *TM6SF2* contribute to disease susceptibility, while *HSD17B13* appears protective [80,81]. In a meta-analysis of 6540 cases and 2,096,759 controls, 10 loci were associated with HCC, including five novel variants, with *PNPLA3* and *TM6SF2* showing the strongest effects; genetic risk closely paralleled steatosis and cirrhosis across metabolic groups [82]. In a Latin American cohort, a composite genetic risk score (*PNPLA3*, *TM6SF2*, *MBOAT7*, *HSD17B13*) was strongly associated with MASLD-related HCC in both cirrhotic and noncirrhotic livers, and linked to distinct immune profiles [83]. Together, these data support integrated genetic risk stratification for identifying high-risk individuals early in MASLD, although clinical application will require combination with clinical and biomarker-based approaches.

### 4.1. Clustering Based on Genetic Polymorphism

In an effort to identify genetic predisposition to hepatic steatosis and subsequent HCC development in noncirrhotic MASLD, gene variants in *PNPLA3*, *TM6SF2*, *GCKR*, and *MBOAT7* were combined into a polygenic risk score (PRS) and adjusted for *HSD17B13* (PRS-5) in a Mendelian randomization analysis of multiple European cohorts [50]. Both PRS and PRS-5 were strongly associated with the development of cirrhosis and HCC, and these associations remained significant after adjustment for cirrhosis [50]. These findings support a causal role of genetically driven steatosis in HCC development that is only partially mediated through fibrosis progression.

In a separate study, GWAS and proteomic analyses of 9491 clinical cases were integrated with proton density fat fraction (PDFF) data derived from 36,116 liver MRI scans in the UK Biobank [51]. This approach identified 18 gene variants associated with steatosis and four associated with cirrhosis. Notably, steatosis-associated variants influenced the risk of cirrhosis and HCC in proportion to their effects on PDFF, supporting a dose–response relationship between genetically predisposed steatosis and advanced liver outcomes [51]. Although the underlying molecular and cellular mechanisms remain incompletely defined, these findings support the view that MASLD-related hepatocarcinogenesis may, in part, be determined at the stage of steatosis. Identification of PRS-based markers that capture a direct link between pre-cirrhotic events and hepatocarcinogenesis may represent an important step toward risk stratification and cost-effective HCC surveillance in selected MASLD populations.

In a recent work, a two-sample MR analysis was performed using genetic data from Biobank Japan (1866 cases and 195,745 controls from East Asia), deCODE genetics (406 cases and 49,302 controls from Europe), and the UK Biobank (168 cases and 372,016 controls from Europe) [84]. The analysis evaluated 22 modifiable risk factors in East Asian populations and 33 in European populations to assess causal associations with HCC across ancestries. In East Asians, alcohol intake (frequency and ever drinking), AST, hypothyroidism, chronic hepatitis B and C, MASLD, and autoimmune hepatitis were significantly associated with increased HCC risk (*p* < 0.05/22). In Europeans, ALT, AST, MASLD, percent liver fat, and liver iron content were significantly associated with increased HCC risk (*p* < 0.05/33) [84].

In a separate study, gene expression patterns associated with MASLD were analyzed across five clinical states (healthy, obese, steatosis, MASH, and HCC) to improve prediction accuracy by distinguishing gene signatures associated with disease development (i.e., healthy to steatosis) from those associated with disease progression (i.e., steatosis to HCC) [85]. Least absolute shrinkage and selection operator (LASSO) regression was used to identify genes involved in transitions between these states. *CYP7A1* and *TNFRSF12A* were significantly associated with progression of steatosis to HCC [85]. However, predicting progression from steatosis to HCC remains highly challenging, and these findings require validation in larger and more robust datasets. Nevertheless, stepwise analysis of differentially expressed genes across disease states in MASLD represents a plausible approach for identifying expression patterns that may serve as biomarkers or therapeutic targets.

### 4.2. Epigenetic Changes and HCC Risk

Epigenetic alterations may also contribute to the development of HCC in noncirrhotic MASLD. Key mechanisms include global DNA hypomethylation of oncogenes and hypermethylation of tumor suppressor genes; histone modifications that alter chromatin accessibility to favor oncogene activation and tumor suppressor silencing; genome-wide chromatin loop rearrangements that promote enhancer–promoter oncogenic interactions; and RNA-based regulation through microRNAs and other RNA modifications [86]. Metabolic risk factors can induce these epigenetic changes, creating a persistent pro-oncogenic landscape that promotes hepatocarcinogenesis even after metabolic conditions improve, a phenomenon termed “metabolic memory” [87]. Notably, reduced global methylation is not observed when comparing normal and cirrhotic liver tissue, suggesting that DNA hypomethylation may not represent an early driver of tumorigenesis, but rather, may reflect later events in malignant transformation [88].

Together, these studies highlight the promise of genetic and genomics-based approaches for refining HCC risk prediction in MASLD and identifying vulnerable subgroups, particularly by capturing pathways linked to steatosis that are not fully explained by fibrosis alone. This may be especially relevant in noncirrhotic MASLD, where early identification of at-risk individuals cannot rely on advanced fibrosis and remains poorly addressed by traditional biomarkers. However, current evidence is still limited, with a need for broader validation across diverse populations and clearer demonstration of incremental predictive value. Accordingly, incorporation of genetic risk scores into routine clinical algorithms remains premature, particularly given cost and other feasibility constraints in surveillance settings.

## 5. Use of Multi-Omics to Define MASLD Clusters for HCC Risk

### 5.1. Liver Tissue-Based Transcriptomic Profiling

More than 15 years ago, genome-wide expression profiling of formalin-fixed, paraffin-embedded tissues showed that survival in HCC is driven not by tumor gene expression but rather by transcriptomic signatures in adjacent non-tumoral liver tissue [89]. These findings led to the development of a hepatic transcriptome-based prognostic liver signature (PLS), which predicts long-term HCC risk in cirrhosis regardless of etiology (HCV, HBV, alcohol, or MASH) [90]. However, the clinical utility of a transcriptome-based PLS has been limited by the need for liver biopsy. To overcome this, a blood-based surrogate termed prognostic liver secretome signature (PLSec) was developed by translating tissue transcriptomic signatures into circulating secretome markers [91]. PLSec combines six high-risk proteins (vascular cell adhesion molecule 1 [VCAM-1], insulin-like growth factor-binding protein 7 [IGFBP-7], gp130, matrilysin, interleukin-6 [IL-6], and C-C motif chemokine ligand 21 [CCL-21]) and two low-risk proteins (angiogenin and protein S). Its predictive performance improves further with the addition of alpha fetoprotein, yielding the PLSec-AFP model. This etiology-agnostic tool has been externally validated in cirrhosis cohorts and distinguishes low-risk and high-risk patients with an at least a two-fold difference in annual HCC incidence (15 per 1000 PY in the low-risk group and 48 per 1000 PY in the high-risk group) [91].

More recently, the PLSec-AFP score was refined to better identify patients with cirrhosis at negligible risk for HCC and who may not benefit from HCC surveillance [92]. In this approach, PLSec was evaluated in combination with established clinical models, including the aMAP (age, male sex, albumin-bilirubin, and platelets) score, the Toronto HCC Risk Index, and the ADRESS (age, diabetes, race, etiology of cirrhosis, sex, and severity) score [92]. PLSec-AFP was ultimately integrated with the best performing clinical model, aMAP, to generate the PAaM score.

In a derivation cohort of 327 patients with cirrhosis, PAaM cutoffs stratified patients into low (<4.32), intermediate (4.32–5.06), and high (≥5.07) risk groups. Validation in two large prospective cirrhosis cohorts, the Texas HCC Consortium (THCCC; *n* = 2516) and the HCC Early Detection Strategy (HEDS; *n* = 1328), confirmed robust risk discrimination. In the THCCC cohort, the PAaM score corresponded to annual HCC incidences of 0.6%, 2.7%, and 5.3%, in the low-, intermediate-, and high-risk groups, respectively. In the HEDS cohort, the corresponding incidences were 0.8%, 1.8%, and 6.2%. In both cohorts, PAaM outperformed PLSec-AFP and aMAP alone [91]. Notably, the PAaM score has not yet been validated in noncirrhotic liver disease, although MASLD was a common etiology of cirrhosis in the validation cohorts.

Separately, immunoglobulin M-free apoptosis inhibitor of macrophage (AIM) has emerged as a promising single biomarker for early HCC prediction in MASLD, with reported sensitivity, specificity, and accuracy of 74.1%, 95.3%, and 87.1%, respectively [93].

Collectively, liver tissue-derived transcriptomic signatures and their blood-based surrogates represent a promising shift toward biologically informed HCC risk stratification, allowing for refined prognostication beyond conventional clinical models. However, broader clinical adoption will depend on validation in noncirrhotic populations, including early-stage MASLD, and demonstration that these approaches meaningfully improve surveillance strategies and patient outcomes.

### 5.2. Metabolomic Profiling

Metabolomic profiling has recently emerged as a strategy to predict risk of malignancies, including HCC. In a prospective, nested case–control study using the Southern Community Cohort Study (SCCS) for derivation and the UK Biobank (UKB) for validation [94], 150 incident HCC cases were identified among 37,175 SCCS participants with baseline plasma samples (median 7.9 years to diagnosis) and compared to 100 cirrhosis controls and 150 matched noncirrhosis controls. Global metabolomic profiling, performed using four complementary ultrahigh-performance liquid chromatography mass spectrometry (LC/MS) platforms, yielded 662 metabolites for analysis. In the UKB, 12 incident HCC cases (median 7.3 years to diagnosis) among 488 participants were compared with 24 matched cirrhosis controls, and 249 metabolite-related entities were measured in the baseline blood samples using NMR spectroscopy [94].

Metabolite set enrichment analysis (MSEA) in the SCCS and UKB cohorts demonstrated enrichment of pathways related to amino acid metabolism (including tyrosine, glycine/serine/threonine, methionine/cysteine, histidine, and branched chain amino acids), sphingolipid metabolism, coenzyme A biosynthesis, and primary bile acid biosynthesis among individuals who later developed HCC [94]. In the SCCS cohort, clinical covariates alone predicted HCC with an area under the curve (AUC) of 0.66, which improved to 0.85 with the addition of a 10-metabolite signature and to 0.88 when combined with the top three clinical predictors (age, alcohol use, and physical activity). In the UKB cohort, inclusion of four overlapping metabolites (glycine, histidine, sphingomyelins, and cholines) increased the AUC from 0.72 to 0.88. Overall, pre-diagnostic metabolites were associated with incident HCC across two demographically distinct cohorts, although their utility in strictly noncirrhotic MASLD populations remains uncertain [94].

In summary, metabolomic profiling highlights the potential of circulating metabolic signatures to improve early HCC risk prediction beyond clinical variables, although their applicability in noncirrhotic MASLD populations has not yet been established.

## 6. Use of Machine Learning to Define MASLD Clusters for HCC Risk

**Figure 3:**
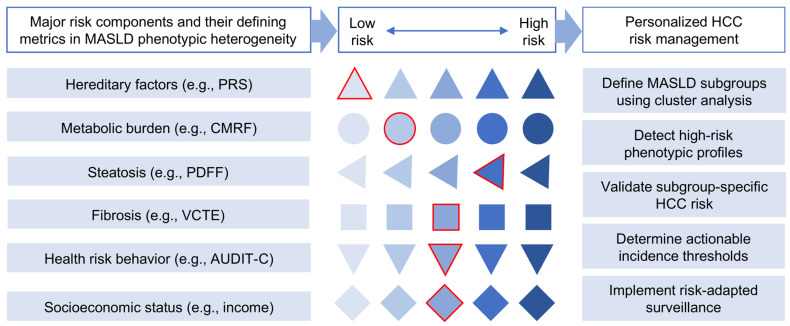
Risk-based identification of MASLD subgroups for early HCC surveillance. Conceptual framework for hepatocellular carcinoma (HCC) risk prediction and development of targeted surveillance in high-risk subgroups in metabolic dysfunction-associated steatotic liver disease (MASLD) using composite scores that integrate genetic susceptibility, clinicopathologic features, and environmental exposures. Within each risk domain, distinct shapes (e.g., triangle, square, circle) illustrate categorical strata ordered from lower to higher risk. Selection of individual features for a hypothetical patient is shown by red outlines, indicating components contributing to an overall composite risk score. Machine-learning approaches may allow for integration of these and additional categorical and continuous variables to define biologically and clinically meaningful MASLD subgroups and generate weighted risk estimates, potentially informing personalized surveillance strategies in selected noncirrhotic patients. AUDIT-C, alcohol use disorder identification test–consumption; CMRF, cardiometabolic risk factor; HCC, hepatocellular carcinoma; MASLD, metabolic dysfunction-associated steatotic liver disease; PDFF, proton density fat fraction; PRS, polygenic risk score; VCTE, vibration-controlled transient elastography.

Phenotypic heterogeneity is a defining feature of MASLD and a major barrier to accurate risk stratification for disease progression and HCC development, particularly in noncirrhotic populations. MASLD heterogeneity makes it essential to identify subgroups at increased risk of developing complications such as noncirrhotic HCC. Machine learning (ML), a rapidly advancing domain within AI, offers powerful tools to analyze high-dimensional datasets and identify latent patterns not readily captured by conventional statistical approaches. In this context, unsupervised ML methods, including clustering techniques, have been increasingly applied to MASLD populations to identify biologically and clinically meaningful subgroups with distinct risks of liver-related and extrahepatic outcomes [95,96,97,98,99,100]. By integrating genetic, clinical, laboratory, imaging, behavioral, and socioeconomic variables, these approaches have identified reproducible MASLD phenotypes with distinct prognostic trajectories [101,102,103,104], underscoring the potential of ML to refine risk estimation beyond fibrosis-based staging alone (Figure 3).

Different ML methods provide complementary advantages in developing predictive models for MASLD progression and complications [105,106]. Interpretable models, such as logistic regression and penalized approaches (e.g., LASSO), are useful for feature selection and downstream risk modeling within identified clusters but may be limited in capturing nonlinear relationships [107]. In contrast, tree-based ensemble methods, including random forests (RF) and gradient boosting, better accommodate complex interactions and heterogeneous data structures, making them well suited for defining and validating phenotypic clusters, albeit with reduced interpretability and potential overfitting [108]. More complex ML approaches, including deep learning (DL) that developed from neural network analysis, are particularly valuable for integrating multimodal and unstructured data but require large datasets and careful validation [109].

Across methods, key limitations include data heterogeneity and missingness, class imbalance, and limited external validation, which may constrain generalizability. In practice, simpler models may facilitate clinical translation; whereas more complex ML approaches may be better suited for identifying high-risk MASLD subgroups, including those at risk for noncirrhotic HCC, that are not captured by conventional frameworks. Importantly, greater model complexity does not necessarily translate into improved predictive performance, and parsimony remains essential when incorporating a large number of biomarkers into ML-based analyses [101,104,110,111].

AI models leveraging electronic health record (EHR) data, including recurrent neural networks and gradient-boosting machines, have demonstrated strong performance in predicting HCC across chronic liver diseases [112]. Notable examples include the PLAN-B gradient-boosting model for chronic hepatitis B [113] and the SMART random survival forest model for HCC prediction following sustained virologic response in hepatitis C [114]. These studies provide proof of concept that ML can integrate longitudinal clinical data to generate individualized HCC risk estimates.

Evidence supporting ML-based HCC prediction is now emerging specifically in MASLD. EHR data from two University of California health systems were used to determine the ability of ML methods to predict the probability of progression from MASLD to HCC [115]. The analysis included patients with baseline anthropometric and clinical parameters collected within the first year of MASLD diagnosis, irrespective of whether they subsequently developed HCC between 2010 and 2021. Five ML algorithms (decision tree, gradient boosting, naïve Bayes, probabilistic neural network, and RF) were evaluated, with gradient boosting demonstrating the best performance (accuracy 92.06%, AUC 0.97, specificity 98.34%, sensitivity 74.41%). The most influential predictors included the FIB-4 index, hypertension, and serum levels of alkaline phosphatase, total cholesterol, and total bilirubin, highlighting the value of combining readily available clinical and laboratory variables within ML frameworks [115]. Notably, the analysis was subject to substantial selection bias, as the proportion of patients at high risk for HCC was 50% in the discovery cohort and 55% in the validation cohort, limiting the generalizability of these findings to the broader MASLD population.

ML approaches integrating multi-omics data further expand the scope of MASLD subgroup identification and HCC risk prediction. Untargeted lipidomics combined with explainable AI have identified lipid signatures associated with liver cancer risk, with AdaBoost models achieving robust discrimination and Shapley Additive Explanations (SHAP) analysis highlighting biologically relevant lipid drivers [116]. Similarly, ML applied to bulk and single-cell transcriptomic data has identified key prognostic genes implicated in MASH-related HCC progression, including *TP53I3* and *SOCS2* [117].

Compared with traditional ML approaches, DL is particularly well suited for analyzing complex, unstructured data, such as radiologic and histopathologic images, molecular networks, and genetic sequences, owing to its use of multilayered neural networks that consist of multiple hidden layers and learn progressively more abstract feature representations [109]. In the context of MASLD, these capabilities have been leveraged for imaging-based steatosis quantification, automated fibrosis staging, radiomics, and digital pathology, representing some of the most advanced AI applications in hepatology [105,106]. In a case–control study of biopsy-proven steatotic liver disease, a DL model predicted future HCC development with an AUC of 0.80 by capturing pathological features beyond fibrosis, including nuclear atypia, altered hepatocyte morphology, immune infiltration, and lipid droplet characteristics [118]. However, the applicability of DL-based approaches at the population level remains constrained, as these methods typically utilize MR imaging or histopathology data that are not commonly available in most individuals with noncirrhotic MASLD.

In summary, these studies highlight the potential of AI-driven approaches to integrate heterogeneous data modalities and capture the biological and clinical complexity of MASLD, enabling the identification of patient subgroups at increased risk of HCC who may benefit from targeted surveillance. While HCC risk prediction in advanced MASLD, largely driven by the presence of advanced fibrosis or cirrhosis, may be incrementally improved by these methods, it is comparatively more straightforward given the availability of established clinical markers. In contrast, the greatest value of AI lies in early-stage MASLD, where risk stratification is poorly defined and likely depends on integrating high-dimensional multi-omics information with clinicopathologic, lifestyle, and socioeconomic data that reflect non-fibrosis-driven mechanisms of hepatocarcinogenesis. Applying HCC risk prediction in this setting must balance potential benefits with harms and costs. Scalable strategies will require initial identification of high-risk groups using accessible biomarkers, followed by advanced analytical approaches. Robust comparative effectiveness data and prospective validation studies will be essential before these approaches can be translated into clinical recommendations or screening strategies.

## 7. Conclusions

Given the high prevalence and marked heterogeneity of MASLD, identifying subgroups at increased risk for HCC in the noncirrhotic setting remains a major clinical and public health challenge. Defining these at-risk subgroups, along with their corresponding HCC incidence, is essential to guide cost-effectiveness analyses and establish evidence-based thresholds that meet current criteria for surveillance recommendations. In the absence of clearly characterized risk strata, implementing targeted screening strategies is currently not feasible. Moreover, existing noninvasive tools, largely designed for fibrosis staging, may be insufficient for HCC risk prediction, particularly given that hepatocarcinogenesis in noncirrhotic MASLD may follow distinct biological pathways. Addressing this gap will require the development of risk models tailored to pre-cirrhotic MASLD that integrate both traditional and emerging biomarkers. Incorporating machine learning approaches in this integration to capture disease heterogeneity may revolutionize risk stratification, ultimately allowing for the identification of clinically meaningful subgroups of non-advanced MASLD for risk-based and cost-effective HCC surveillance.

## Figures and Tables

**Table 1:** Demographic and metabolic contributors to HCC risk independent of fibrosis: evidence from large cohort studies.

Authors/Year	Study Design and Population	Key Findings
Kanwal et al., 2020 [35]	Retrospective cohort study of 271,906 individuals with MASLD within the US Veterans Health Administration (VHA), enrolled between 2004 and 2008 and followed for a median of 9 years through 2015	Approximately 25% of HCC cases occurred in the absence of cirrhosis. Hispanic ethnicity, older age, and male sex were associated with the composite outcome of cirrhosis or HCC. Metabolic comorbidities independently increased HCC risk, with T2D showing the strongest association (2.77-fold higher risk). The presence of multiple metabolic risk factors conferred progressively higher HCC risk
Ebrahimi et al., 2023 [46]	Nationwide multigenerational retrospective cohort study in Sweden including 11,909 individuals with biopsy-confirmed MASLD (biopsied between 1969 and 2017) and 56,512 matched population controls	First-degree relatives of individuals with MASLD had a 70–80% higher HCC risk than the general population, particularly when the index case had fibrosis or cirrhosis (aHR 2.14). Although spouses did not show a statistically significant increase in HCC risk, they had higher rates of adverse liver outcomes and liver-related mortality; T2D emerged as a strong independent predictor of HCC among spouses (aHR 10.5)
Lai et al., 2024 [33]	Retrospective nationwide cohort study using a US administrative claims database, including 741,816 individuals with SLD followed from 2007 to 2021	Among 741,816 patients, 14.7% had cirrhosis. Over 2.41 million PY of follow-up, 1740 HCC cases occurred (0.72 per 1000 PY). HCC incidence was markedly higher in patients with cirrhosis compared to those without (4.29 vs. 0.14 per 1000 PY) and was further associated with older age, male sex, T2D, CKD, CVD, and non-liver malignancy. Among noncirrhotic patients, males >70 years with T2D had an incidence of 1.36 per 1000 PY
Kim et al., 2024 [47]	Retrospective cohort study in Korea including a nationwide cohort of 409,088 individuals and a hospital-based cohort of 8721 individuals with noncirrhotic MASLD, followed for a median of 10 years	Six independent risk factors for HCC in noncirrhotic MASLD were identified, including age, sex, diabetes, obesity, and serum levels of ALT and GGT
Cho et al., 2025 [48]	Retrospective population-based cohort study in Korea including 92,761 adults aged 40–79 years with T2D who underwent health screening between 2012 and 2015, with incident HCC identified from 2016 to 2020	Both baseline FLI and longitudinal changes influence HCC risk: rising FLI is associated with higher risk, whereas declining FLI is linked to risk reduction. Notably, the protective effect of FLI improvement becomes evident after ~3 years, suggesting that sustained metabolic improvement may translate into meaningful long-term oncologic benefit
John et al., 2026 [45]	Retrospective cohort study of 666,428 participants from the Veterans Analysis of Liver Disease (VALID) cohort, including individuals with MASLD, MetALD, ALD, and non-steatotic liver disease controls, followed from 2011 to 2023	MASLD and MetALD had comparable 5-year cumulative HCC incidence (450 vs. 451 per 100,000). By 10 years, MetALD showed a modestly higher incidence than MASLD (815 vs. 763 per 100,000), likely reflecting the added contribution of alcohol exposure
Chun et al., 2026 [42]	Multinational, multicenter retrospective cohort study including 77,677 individuals with MASLD enrolled 2003–2014, comprising 73,599 participants from five tertiary hospitals in Korea and 4078 participants from 15 centers across 11 countries including China, Japan, Italy, France, and the US	Several factors were independently associated with HCC risk, including overweight/central obesity, prediabetes or T2D, older age, male sex, and platelet count

aHR, adjusted hazard ratio; ALD, alcohol-related liver disease; ALT, alanine aminotransferase; ASCVD, atherosclerotic cardiovascular disease; AST, aspartate aminotransferase; CKD, chronic kidney disease; CVD, cardiovascular disease; FLI, fatty liver index; GGT, gamma-glutamyl transferase; HCC, hepatocellular carcinoma; MASLD, metabolic dysfunction-associated steatotic liver disease; MetALD, metabolic dysfunction and alcohol-associated steatotic liver disease; PY, person-year; SLD, steatotic liver disease; T2D, type 2 diabetes.

## Data Availability

No new data were created or analyzed in this study. Data sharing is not applicable to this article.
